# Analysis of tilianin and acacetin in *Agastache rugosa* by high-performance liquid chromatography with ionic liquids-ultrasound based extraction

**DOI:** 10.1186/s13065-016-0223-7

**Published:** 2016-11-29

**Authors:** Jinfeng Wei, Pengran Cao, Jinmei Wang, Wenyi Kang

**Affiliations:** 1 Institute of Chinese Materia Medica, Henan University, Kaifeng, 475004 China; 2 Kaifeng Key Laboratory of Functional Components in Health Food, Kaifeng, 475004 China

**Keywords:** Ionic liquids-ultrasound, HPLC, *Agastache rugosa*, Tilianin, Acacetin

## Abstract

Ionic liquid 1-butyl-3-methylimidazolium bromide-methanol-based ultrasonic-assisted extraction (ILUAE) was used to extract tilianin and acacetin from the aerial parts of *Agastache rugose* (*A. rugose*), and simultaneously determined by reversed phase high performance liquid chromatographic (RP-HPLC) method with ultraviolet detection (RP-HPLC-UV). An InertSustain RP-C_18_ column was used with the mobile phase consisting of methanol and 0.2% acetic acid as gradient elution at the detection wavelength of 332 nm. The flow rate was 0.8 mL/min, and the column temperature was 30 °C. Under the optimized conditions, tilianin and acacetin displayed good linearity in the ranges of 0.0595–4.76 and 0.0585–4.68 μg/mL, respectively, with the average recoveries being 96.93 and 97.88%, respectively. The method of ILUAE was compared with the traditional methods, it exhibited higher efficiency, higher reproducibility and environmental friendly in analyzing the active compounds in traditional Chinese medicines (TCMs).

## Background

*Agastache rugosa* (Fisch. & C.A.Mey.) Kuntze (*A. rugose*), a medicinal plant belonging to the family *Lamiaceae,* is native to China, Korea, and Japan. *A. rugosa* shows the similar taste and thermal properties to those of pungent (acrid) and is slightly warm with the channel affiliations entering spleen, stomach and lung. It can dispel damp, and relieve nausea and vomiting, and cure fungal infections. Pharmacological investigations have shown that *A. rugosa* have antiviral [1], antimicrobial [2], antioxidant [3], cardiovascular and anti-inflammatory [4, 5] activities, and some other activities [6].

At present, there are still many problems during the process of development and utilization of TCM resource, including lower efficiency, higher energy consumption, higher pollution, longer production cycle, and waste of resources etc. The products of TCMs also have many shortcomings, for example, low yield, many impurities and poor quality etc [7]. Ionic liquid (IL) is a new substance which has been developed in the framework of green chemistry in the recent years. Ionic liquids (ILs) are the substances solely composed of anions and cations. The interests in the ILs have significantly increased because of their special properties. They are good solvents for both organic and inorganic liquids, over a wide range of temperatures, and are not volatile, highly negligible vapor pressure, thermally stable, nonflammable, polar, weakly coordinating solvents and less toxic than usual organic solvents [8, 9]. Using IL as a solvent to extract the active ingredients of TCM is not only environmental friendly, but also selective and also has high yield and pre-concentration [10]. Thus, the extraction of active ingredients of TCM by IL is a breakthrough method, because it provides reference for the healthy and sustainable development of TCM resources.

The conventional methods of extraction of natural products from plant materials are mainly by maceration, which is very time-consuming and requires relatively large quantities of toxic organic solvents [11]. Ultrasonic-assisted extraction (UAE) has been found to be a more effective and environmentally friendly way of extracting natural product from plant materials for its characteristics of shorter extraction time and use of less amounts of organic solvents. Several groups of investigators extracted the total flavonoids from corn silk by UAE and obtained a high extraction yield [12–17]. Thus, in this study, we chose UAE to extract the target analytes from *A. rugose*.

In our previous chemical research, we isolated abundant tilianin and acacetin from *A. rugosa*. Our previous studies together with the other reports [4, 18] have indicated that tilianin and acacetin are the main active compounds. Thus, 1-butyl-3-methylimidazolium bromide-methanol solution was used as extraction agent in combination with high performance liquid chromatography (HPLC) simultaneous to separate and determine tilianin and acacetin. To our best knowledge, the simultaneous extraction of flavonoid and flavonoid glycosides in *A. rugosa* using IL has not been reported yet to date. The present study aimed to establish a rapid, greener and effective ionic liquid-based ultrasonic-assisted extraction method (ILUAE) for simultaneous extraction of tilianin and acacetin from *A. rugosa*.

## Experimental methods

### Chemicals and material

Methanol of chromatographic grade was purchased from Tianjin Da Mao Chemical Reagent Factory (Tianjin, China). The ultra pure water was purchased from Hangzhou Wahaha Baili Food Co. Ltd, (Zhejiang, China). Acetic acid was obtained from Tianjin Fu Chen Chemical Reagent Factory (Tianjin, China). 1-butyl-3-methyl imidazolium tetrafluoroborate ([BMIM]BF_4_), 1-butyl-3-methyl imidazole bromide ([BMIM]Br) and 1-butyl-3-methylimidazoliumhexafluorophosphate ([BMIM]PF_6_) were obtained from limited partnership Merck (Darmstadt, German). 1-hexyl-3-methylimidazolium hexafluorophosphate ([HMIM]PF_6_) was purchased from Thermo Fisher Scientific (Rockville, MD, USA).

A LC-20AT high performance liquid chromatography system (Shimadzu, Kyoto, Japan), equipped with a degasser, a quaternary gradient low pressure pump, the CTO-20A column oven, a SPD-M20AUV-detector, a SIL-20A auto sampler was used. Chromatographic separations of target analytes were performed on an InertSustain C_18_ column (4.6 mm × 250 mm, 5 µm). KQ-500DB ultrasonic cleaner (Jiangsu Kunshan Ultrasonic Instrument Co., Ltd. Jiangsu, China); FZ102 micro plant sample pulverizer was obtained from Huanghua, Hebei Zhongxing Instrument Co., Ltd. (Baoding, Hebei, China); sample sieves were obtained from Sieve Factory (Five Four instrument, Shangyu, Zhejiang, China); AB135-S 1/10 million electronic balance was purchased from Mettler Toledo Instruments Co., Ltd. (Shanghai, China).

### Plant material and sample preparation

*Agastache rugosa* (Fisch. & C.A.Mey.) Kuntze (*A. rugose*) was collected in October 2013 from the Suzhou region of Jiangsu Province, China and identified by a plant scientist, Professor Changqin Li. A voucher specimen (201310231) was deposited in the Institute of Traditional Chinese Medicine, Henan University. The plants were dried in shade at room temperature, the dried plants material were pulverized and then passed successively through 90, 70, 50, 24 and 10-mesh sieves.

For sample solution, 50 mg of *A. rugosa* powder was passed through 50-mesh sieve, and put in 1.5 mL centrifuge tube. 1 mL of the extraction solution was added, followed by ultrasonic extraction for 30 min to obtain supernatant. The supernatant was passed through a 0.22 μm organic microporous membrane. The filtrate was obtained and used as the sample solution.

For standard sample solution, 1.17 mg of acacetin and 1.19 mg of tilianin were dissolved in methanol in 25 mL volumetric flask to yield the stock solutions. The concentrations were 0.0468 and 0.0476 mg/mL, respectively.

### Ionic liquids-based ultrasonic-assisted extraction

The preparation steps of ILs methanol solutions were as follows (0.8 M): certain amounts of different ionic liquids (according to their molar masses) were accurately weighed, fully dissolved in methanol and then diluted to 1 mL with methanol in a volumetric flask (1 mL), respectively. 50 mg of dried sample powder was mixed well with 1 mL of IL-methanol solution in a 1.5 mL centrifuge tube. The centrifuge tube was then most partially immersed into the ultrasonic water bath. The temperature of water bath was controlled by the replacement between inlet and outlet water. The bath power rating was 100 W. At room temperature (20 °C), after ultrasonic extraction, the contents of tilianin and acacetin were determined by reversed phase high performance liquid chromatographic-ultraviolet (RP-HPLC-UV). The type of ILs, the concentration of selected IL, the mesh sieve through which of *A. rugosa* was passed, the ultrasonic time and solid–liquid ratio were systematically investigated in this experiment.

### Chromatographic conditions

Chromatographic conditions were set as follows: separation column, InertSustain C_18_ column (4.6 mm × 250 mm, 5 μm); mobile phase, methanol (B)-0.2% aceticacid (C), gradient elution (0–10 min, 40–55%B, 60–45%C; 10–20 min, 55–65%B, 45–35%C; 20–30 min, 65–75%B, 35–25%C; 30–40 min, 75–80%B, 25–20%C; 40–50 min, 80–100%B, 20–0%C; 50–70 min, 100%B), column temperature, 30 °C; flow rate, 0.8 mL/min; the UV detection wavelength, 332 nm; and sample volume, 10 μL.

## Results and discussion

### The selection of the wavelength

Xie et al. [19] found that the optimum wavelength of acacetin was 332 nm with DAD detector scanning. The optimum wavelength of tilianin was mostly selected at 330 nm [20, 21]. In the experiment, the 332 nm was selected as the detection wavelength because we found that there was less interference at this wavelength.

### Selection of dispersing agent

At room temperature, IL is a liquid with high-viscosity (usually higher than that of the conventional organic solvent by 1–3 orders of magnitude). Because [BMIM]Br is crystalline, we need a suitable solvent to dissolve the IL. During the course of the study, we found that [HMIM]PF_6_ and [BMIM]PF_6_ were water insoluble. The extraction rates of IL-ethanol and IL-acetonitrile were lower than that of IL-methanol, while acetonitrile had a higher toxicity. Thus, methanol was chosen as the dispersing agent.

### Linear relationship

For preparing standard sample solutions, various amounts of tilianin and acacetin were dissolved in methanol to yield the stock solutions, respectively. Corresponding calibration curves for tilianin and acacetin were Y = 1336560814x + 17243, (*r* = 0.9999) and Y = 5785424072x + 27367, (*r* = 0.9999), respectively. Both tilianin and acacetin displayed good linearity in the ranges of 0.0595–4.76 μg/mL and 0.0585–4.69 μg/mL, respectively. The limit of detection (LOD) and the limit of quantification (LOQ) of tilianin were 1.59 and 2.18 ng/mL, respectively while LOD and LOQ of acacetin were 0.1081 and 0.225 ng/mL, respectively.

### Optimization of extraction conditions

#### Type of the ILs determination

**Fig. 1:**
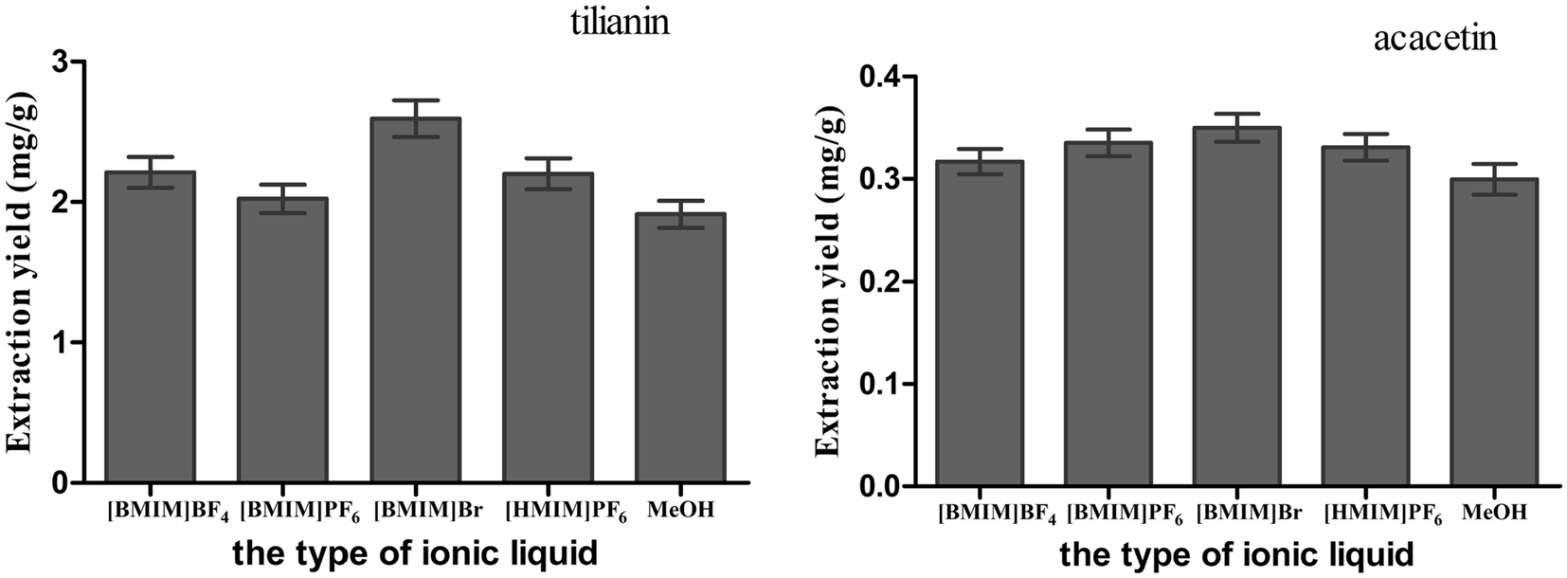
Effect of the type of ionic liquid. Extraction process was performed in an ultrasound unit with a power of 100 W and the concentration of each ILs methanol solution was 0.8 mol/L. 50 mg of the *A. rugosa* powder of passing through 50-mesh sieve, solid–liquid ratio 1:20 (g/mL), ultrasonic for 30 min. The extraction yield was expressed as the observed values of target analytes (mg/g), the content of tilianin and acacetin per gram of *A. rugosa* powder

The structure of ILs had significant influence on their physicochemical properties, which might greatly affect the extraction yields of target analytes [22]. In this experiment, [BMIM]BF_4_, [BMIM]Br, [BMIM]PF_6_, and [HMIM]PF_6_ ILs-methanol were selected as extraction solutions to measure the contents of tilianin and acacetin. The results were shown in Fig. 1. In Fig. 1, the extraction yields of 4 kinds of ILs-methanol solution were higher than that of MeOH. Among these ILs-methanol solutions tested, the extraction yield of [BMIM]Br-methanol was the highest one. Thus, the [BMIM]Br-methanol was chosen as the extraction solution. Ha et al. reported that many ILs showed high capacity for cellulose dissolution, especially halide and phosphate anions [23]. The primary cell wall of medicinal plants is made primarily of cellulose. The ILs mainly action cell wall components, dissolve them in turn, increase the cell wall permeability, resulting in higher yield of the effective constituents [24]. In addition, IL had a good ability to dissolve inorganic and organic matters. The solubility of tilianin and acacetin were not good, and ILs had a good ability to dissolve tilianin and acacetin. Thus, the extraction yields of 4 kinds of ILs methanol solution were higher than that of MeOH.

Four different types of ILs had different effects on the extraction yields of tilianin and acacetin. The types of ILs could influence the extraction yield of target analytes. The ion type and alkyl chain length had an effect on the extraction yields of alkaloids [10]. In this experiment, the effect of type of the IL on extraction yields of flavonoids was not investigated because of the limited types of ILs. Thus, the effect of the type on the extraction rate of flavonoids should be investigated in the subsequent work.

#### Effect of concentrations of the ILs selected

**Fig. 2:**
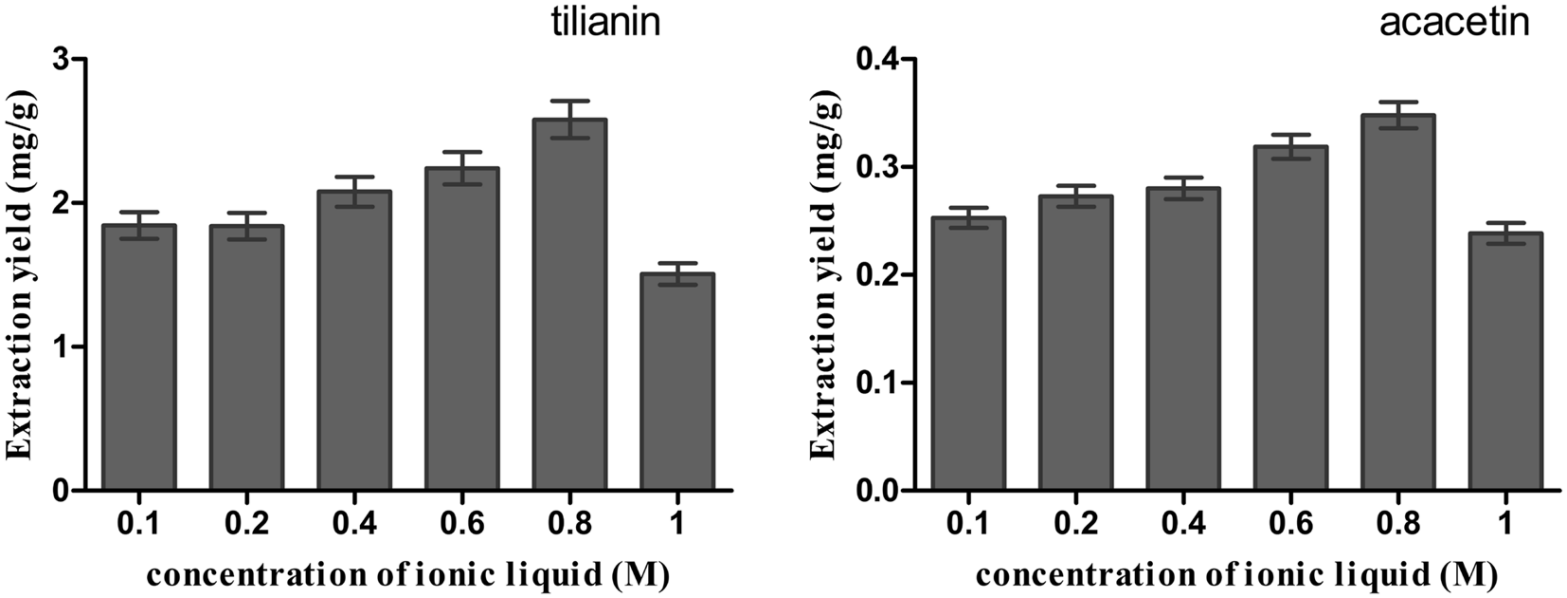
Effect of concentration of the ionic liquid selected. Effect of ionic liquid concentration on the extraction yields of two target analytes, with *A. rugosa* powder of passing through 50-mesh sieve, solid–liquid ratio 1:20 (g/mL), ultrasonic for 30 min. The extraction yield was expressed as the observed values of target analytes (mg/g), the content of tilianin and acacetin per gram of *A. rugosa* powder

Different concentrations of IL had effect on extraction yields of two target analytes. In order to find out the optimal ionic liquid concentration for two target analytes in *A. rugosa*, different concentrations ranging from 0.1 to 1 M of [BMIM]Br-methanol solution were investigated while the other conditions were unchanged. The results were shown in Fig. 2 from which, it can be seen that the extraction yields of the target compounds were gradually increased when the concentration of IL was increased from 0.1 to 0.8 M. With the increase in concentration of IL from 0.1 to 0.8 M, the interactions among IL and *A. rugosa* matrix and cellulose were enhanced, while the dissolution rates of tilianin and acacetin in *A. rugosa* were accelerated and the extraction yields were improved. While in 1.0 M, the extraction yield was decreased sharply. This might be related to the higher viscosity of ILs. The viscosity of IL was much higher than those of water and conventional organic solvent, thus, the mass transfer resistance was larger than that of traditional extracting agent [7]. The high viscosity of the solvent at high concentrations (1.0 M) of IL could lead to poor infiltration of the solvent into the plant tissue and the decreased extraction yields of two target analytes. Based on these results, 0.8 M [BMIM]Br was finally selected for the following experiments.

#### Effect of size of mesh sieves through which *A. rugosa* was passed

It is well known that the size of the crushed particles influences the extraction yield in the process of natural medicine extraction. According to the above experimental methods, the impact of mesh sieves on extraction yield was investigated with 0.8 M [BMIM]Br IL-methanol solution while the other conditions were unchanged.

**Fig. 3:**
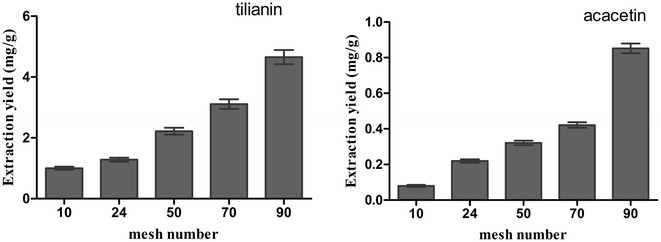
Effect of mesh sieve that *A. rugosa* passing through. Effect of mesh sieve that *A. rugosa* passing through on the extraction yield of two target analytes, with 0.8 mol/L [BMIM]Br, solid–liquid ratio 1:20 (g/mL), ultrasonic for 30 min. The extraction yield was expressed as the observed values of target analytes (mg/g), the content of tilianin and acacetin per gram of *A. rugosa* powder

The results in Fig. 3 showed that the extraction yields of two target analytes became higher and higher when the sizes of particles were smaller and smaller. According to the optimal comminution granularity of TCM [25], the crushing granularity of *A. rugosa* should be from 1 to 4 mm. When the particles were too small, it was hard to filter them. Putting all together, the extraction efficiency of the *A. rugosa* that was passed through 10-mesh sieve to 90-mesh sieve was investigated, and the 90-mesh sieve was ultimately chosen as the optimal condition.

During the process of crushing sample, we found that the crude stem parts of *A. rugosa* had a higher degree of lignification and were hard to shatter while the parts of tender stem and leaves were easily crushed. Thus, with the increase in mesh number, the particles of *A. rugosa* became smaller and smaller and contained more tender stem and leaf parts. It could be inferred that the contents of tilianin and acacetin in the different parts of *A. rugosa* would be quite different.

In classical prescription on TCM, the stem of *A. rugosa* was mostly used to treat diseases of stomach and intestines. However, from the other reports [4–6, 18], we know that tilianin and acacetin didn’t have any activity in these parts. Thus, if we want to clarify the application aspects, we need to reduce the dosage and increase efficacy of drugs. Thus, a lot of work still needs to be continued. The main chemical constituents of different parts of *A. rugosa* need to be identified and the effects of different parts need to be examined in the subsequent work. The contents of active ingredients in *A. rugosa* in different parts need to be identified as well.

#### Effect of ultrasonic time

**Fig. 4:**
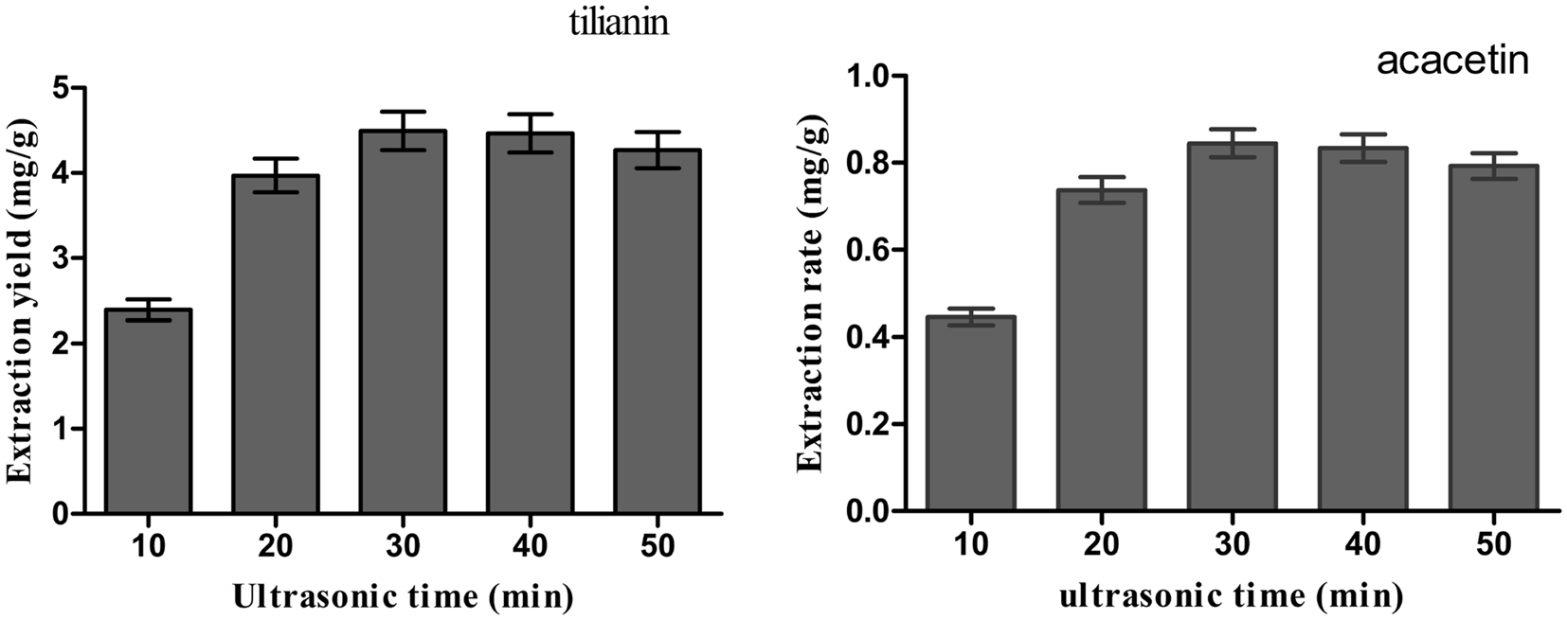
Effect of ultrasonic time. Effect of ultrasonic time on the extraction yield of two target analytes, with *A. rugosa* powder of passing through 90-mesh sieve, 0.8 M [BMIM]Br, solid–liquid ratio 1:20 (g/mL). The extraction yield was expressed as the observed values of target analytes (mg/g), the content of tilianin and acacetin per gram of *A. rugosa* powder

In this study, we observed that ultrasonic time was another leading factor influencing the extraction efficiencies to certain extend. Figure 4 illustrated that the extraction efficiencies of two target analytes were increased with extending the ultrasonic time from 10 to 30 min, but when ultrasonic time reached 40 min, the extraction efficiency were decreased. Thus, ultrasonic time for 30 min was set for further optimization experiments.

The results showed that the extraction yields of the two target analytes were increased with prolonging the ultrasonic time from 10 to 30 min. To extract tilianin and acacetin from the cellular compartments, the solvent must have access to the cellular compartments where the tilianin and acacetin are located. An intact cellular structure restricts accessibility of the solvent to the tilianin and acacetin while ultrasound treated-cells had a more open, fragmented structure, which can facilitate an efficient extraction [22]. The extraction yield reached the maximum when the ultrasonic time was increased to 30 min, while the extraction yield was decreased when the ultrasonic time continued to increase. This may be due to the reason that with the increase of ultrasonic time, some carbohydrate and protein in *A. rugosa* were extracted, the viscosity of the solution is increased and thus, tilianin and acacetin are adsorbed on solid substrate and not easy to be extracted and thus, the extraction yield was decreased [26]. Because of this reason, the ultrasonic time for 30 min was chosen as the optimal condition in this experiment.

#### Effect of solid–liquid ratio

**Fig. 5:**
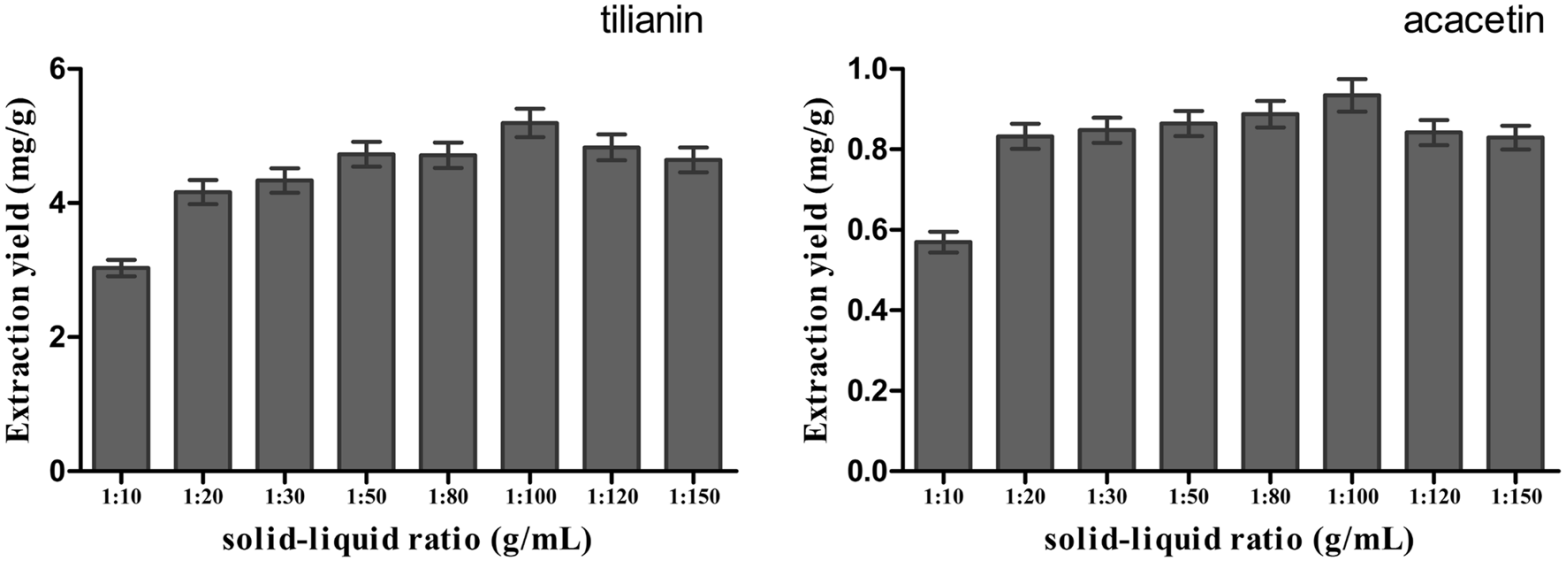
Effect of solid–liquid ratio. Effect of solid–liquid ratio on the extraction yield of two target analytes, with *A. rugosa* powder of passing through 90-mesh sieve, 0.8 M [BMIM]Br, ultrasonic for 30 min. The extraction yield was expressed as the observed values of target analytes (mg/g), the content of tilianin and acacetin per gram of *A. rugosa* powder

To some extent, the solid–liquid ratio was an important parameter, which should be studied to increase the extraction efficiency of two target analytes. On the basis of the above optimized conditions, the effect of solid–liquid ratios on the extraction yield of target extract was investigated. The results were shown in Fig. 5. In Fig. 5, when the solid–liquid ratio was 1:100, the extraction yield reached a maximum. While when the ratio of solid–liquid continued to increase, the extraction yield tended to decline. The dissolution rates of tilianin and acacetin had reached the maximum values at the solid–liquid ratio of 1:100. When the ratio of liquid–solid was further increased, the extraction yield was decreased with the influence of the IL’s properties.

Figure 5 illustrated that when the ratio of solid–liquid was increased from 1:10 to 1:20, the extraction yield was increased sharply. While when the ratio of liquid–solid was increased from 1:20 to 1:100, the extraction yield was increased slowly. Superfluously, higher solid–liquid ratio could cause procedures more complex and the unnecessary waste, while lower ones would make the extraction of targets incomplete and thus, extraction efficiency lower. Therefore, in the large-scale production of industrialization, the ratio of solid–liquid in the range of 1:20 to 1:100 could be selected to save resources. But in this experiment, 1:100 was chosen for the ratio of solid–liquid, because the test was carried out under the optimal conditions.

### Comparison of ILUAE approach with the traditional methods

**Fig. 6:**
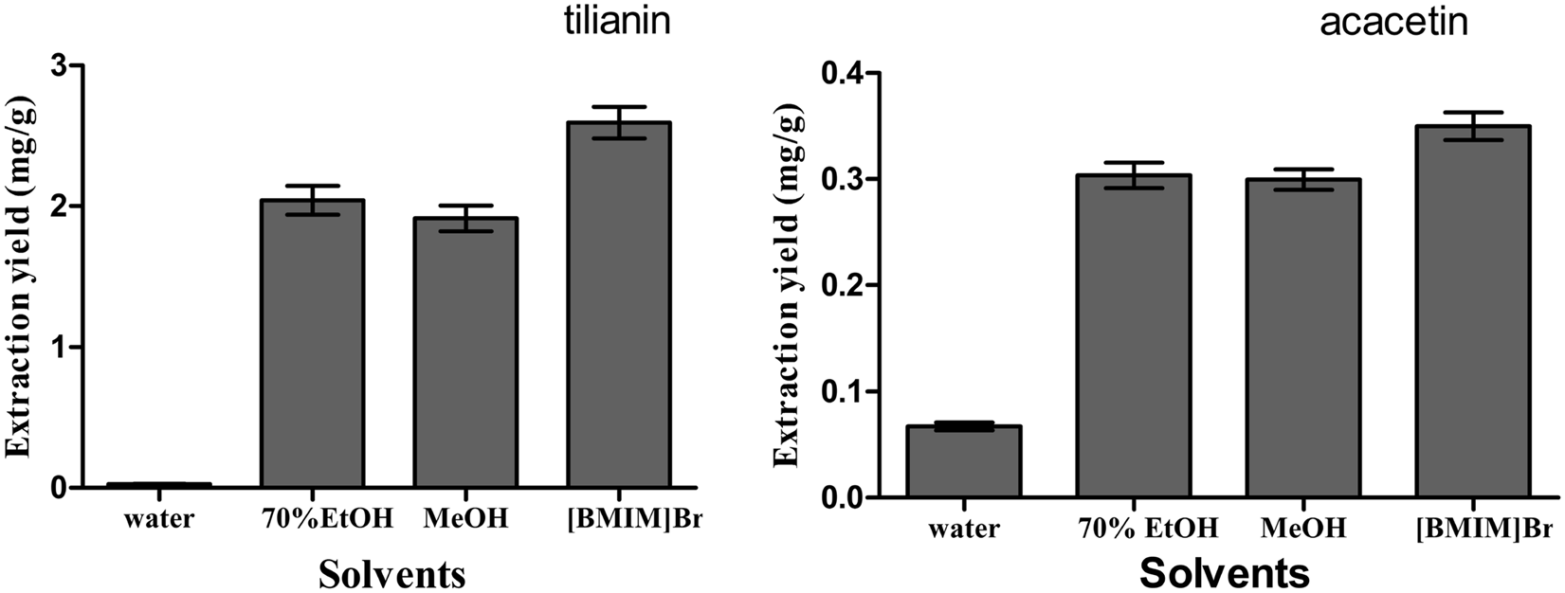
Effect of solvents on the extraction. Effect of the type of solvents on the extraction yield of two target analytes, with *A. rugosa* powder of passing through 50-mesh sieve, 0.8 M [BMIM]Br, solid–liquid ratio 1:20 (g/mL), ultrasonic for 30 min. The extraction yield was expressed as the observed values of target analytes (mg/g), the content of tilianin and acacetin per gram of *A. rugosa* powder

For the solvent extraction frequently used to extract active ingredient from the TCMs, the solvents included pure water and ethanol with different concentrations. Water was the most frequently used as solvent in clinical application of TCMs. In this study, water, 70% ethanol (EtOH) and methanol (MeOH) were used to extract tilianin and acacetin. In Fig. 6, the IL had a higher extraction yield than did the other two methods. Figure 6 illustrated that the proposed approach obviously increased the extraction yield, indicating that the [BMIM]Br solution is an excellent extractant and that ILUAE is a more rapid and effective sample preparation method.

Figure 6 showed that the yields of using water to extract tilianin and acacetin were very low. While the water decoction of *A. rugosa* is always used in clinic, it was speculated that tilianin and acacetin were not the active components in the traditional application. Nevertheless, tilianin and acacetin possess many biological activities but these activities couldn’t find in *A. rugosa*. Interestingly, through our study, we found that tilianin and acacetin had a significant anticoagulant activity, while the 70% EtOH extract of *A. rugosa* didn’t have this activity. Thus, further research is needed to find out the relation between *A. rugosa* and its main components.

### Verification tests

#### Determination of sample

Under the optimal conditions, the powder of *A. rugosa* was passed through 90-mesh sieve, and extracted with 1 mL of 0.8 M [BMIM]Br in 1:100 of solid–liquid, after 30 min of ultrasonic-aided extraction, extraction solution was obtained. The concentrations of tilianin and acacetin in sample solution were measured to be 0.0093 and 0.0529 mg/mL, respectively.

#### Precision experiment

The standard sample solution was determined 6 times according to the above chromatographic conditions. The results showed that the precision of the instrument was good with calculated relative standard deviation (RSDs) values of 0.12 and 0.08%, respectively.

#### Repeatability

**Fig. 7:**
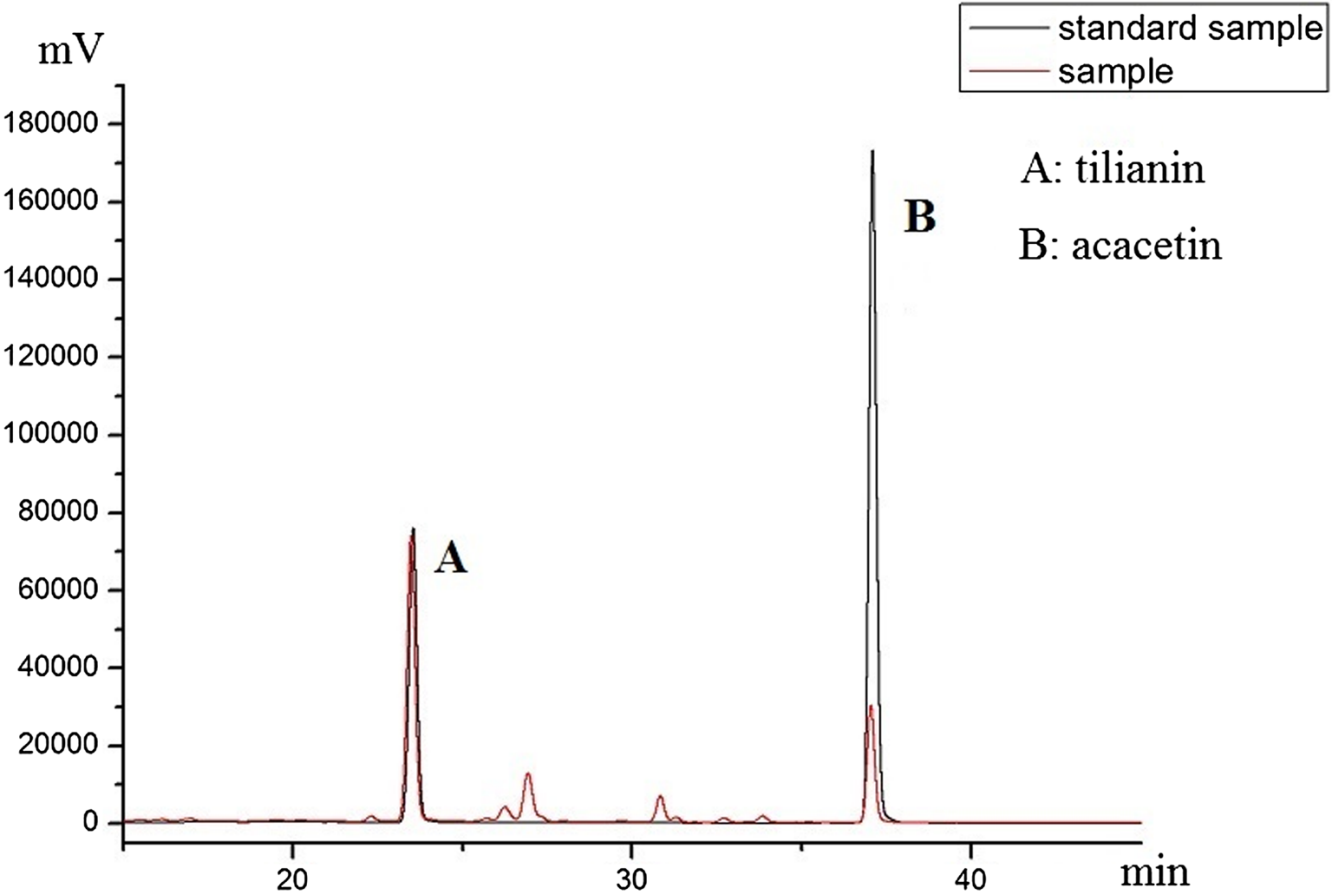
The comparison of sample with standard sample

HPLC chromatograms of the standards solution and sample (under the optimal conditions) are shown in Fig. 7. To determine the repeatability of the novel extraction method, six samples of the same weight (10 mg) were processed under the optimum extraction conditions. The mean extraction efficiencies of tilianin and acacetin obtained under the optimized conditions showed good repeatability with calculated RSD values of 4.02 and 3.90%, respectively. These results indicate that the proposed ultrasound-assisted extraction method has an acceptable level of repeatability.

The results suggest that tilianin and acacetin are stable in the ionic liquid solution during the extraction process. Validation studies on these methods indicate that the proposed method is credible.

#### Stability

The stability of the target analytes under the experimentally derived optimum conditions was assessed by subjecting standards of tilianin (0.0476 mg/mL) and acacetin (0.0468 mg/mL). The recoveries of the target analytes were assumed to be indicative of the stability of the target analytes under the extraction conditions used.

Under the operating extraction conditions, the contents of tilianin and acacetin varied from 100 to 101.8% and from 100 to 100.6%, respectively, within 24 h. The structures of target analytes were stable, with no change in retention time. Therefore the structural change was not significant under the selected optimum conditions.

#### Recovery

Under the optimized conditions detailed above, two samples spiked with tilianin and acacetin were extracted and the recoveries of tilianin and acacetin from dried *A. rugosa* were 96.9 and 97.9%, respectively.

## Conclusions

In this study, an efficient method was developed for the extraction of tilianin and acacetin from *A. rugosa*. The optimum conditions for ILUAE were determined. Compared with traditional methods, the present approach obtained higher extraction yields of tilianin and acacetin, which were 2 to 202 times and 3 to 14 times of those of traditional methods, respectively. This study also demonstrated that the IL solution was an excellent extractant and that ILUAE was a simple, rapid, and effective extraction method. Moreover, with the unique characteristics of IL, the proposed approach in this study had the environmentally friendly, convenient, efficient characteristics and could be the high practical value technique in sample preparation and analysis. Thus, this experiment in combination with the related reports indicates that the extraction of active ingredients in TCM by ionic liquid is a breakthrough one. It provides a theoretical basis for the healthy and sustainable development of TCM resources.

## Abbreviations

ILUAE: ionic liquid based ultrasonic-assisted extraction
TCM: traditional Chinese medicine
HPLC: high-performance liquid chromatography
IL: ionic liquid
ILs: ionic liquids
UAE: ultrasonic-assisted
[HMIM]PF_6_: 1-hexyl-3-methylimidazolium hexafluorophosphate
[BMIM]BF_4_: 1-butyl-3-methyl imidazolium tetrafluoroborate
[BMIM]Br: 1-butyl-3-methyl imidazole bromide
[BMIM]PF_6_: 1-butyl-3- methylimidazolium hexafluorophosphate
LOD: the limit of detection
LOQ: the limit of quantification
EtOH: ethanol
MeOH: methanol
RSD: relative standard deviation
SD: standard deviation
